# Transcranial magnetic stimulation in late-aged people with depressive disorders

**DOI:** 10.1192/j.eurpsy.2023.539

**Published:** 2023-07-19

**Authors:** P. Liubov, D. Egorova

**Affiliations:** ^1^Department of gerontopsychiatry, Moscow Research Institute of Psychiatry - branch of the V. Serbsky National Medical Research Center for Psychiatry and Narcology, Moscow, Russia, Moscow, Russian Federation

## Abstract

**Introduction:**

One of the most common mental disorders in the elderly is depression.

Because of the high frequency of side effects of pharmacotherapy and the comorbid medical illnesses, there are not many ways to treat it.

Non-drug therapies, such as repetitive transcranial magnetic stimulation (rTMS), could help overcome the limitations of standard drug therapy for this type of mental disorders.

**Objectives:**

Development of approaches to improving improve the provision of psychiatric care to elderly patients using rTMS.

**Methods:**

30 patients over the age of 60 with anxiety-depressive spectrum disorders meeting criteria F30-39, F06.3, F06.4 (ICD-10) and a control group with similar criteria that were not treated with rTMS, were recruited from the psychiatric department at a university hospital (Moscow Scientific Research Institute of Psychiatry). Clinical, psychopathological, anamnestic, psychometric (Montgomery-Asberg scale (MADRS), Hamilton scale (HARS), Mini-mental state examination scale (MMSE) instrumental (electroencephalography) research methods were used. Patients of the experimental group underwent 15 sessions of low-frequency rTMS on the right dorsolateral prefrontal cortex (RDLPC). Conditions for the application of 1200 pulses were as follows: frequency - 1 Hz; intensity - 120% of the threshold of motor response (RMT) of the subject; pulse number - 1200; pulse sequence - 300; sequence duration - 300 seconds; sequence interval - 60 seconds; and stimulation time - 23 minutes. Subsequently, the patients were re-examined using the above-mentioned scales to assess their mental state in dynamics.

**Results:**

Analysis of the collected data shows an increase in the number of respondents and the frequency of achieving remission in the experimental group compared to the control group. No severe side effects of rTMS were observed.

**Conclusions:**

rTMS may be a safe method of adjuvant therapy in groups of elderly patients with anxiety-depressive spectrum disorders. Further studies will be needed to clarify the results.

**Disclosure of Interest:**

None Declared

